# Recycled Fine and Coarse Aggregates’ Contributions to the Fracture Energy and Mechanical Properties of Concrete

**DOI:** 10.3390/ma16196437

**Published:** 2023-09-27

**Authors:** Madumita Sadagopan, Alexander Oliva Rivera, Katarina Malaga, Agnes Nagy

**Affiliations:** 1 Department of Resource Recovery and Building Technology, University of Borås, 50190 Borås, Sweden; alexander.oliva.rivera@ri.se (A.O.R.);; 2 RISE-Research Institutes of Sweden, 50115 Borås, Sweden

**Keywords:** fracture mechanics, recycled fine aggregates, recycled coarse aggregates, climate-reduced concrete, eco-concrete, mechanical preprocessing, accelerated carbonation

## Abstract

This paper investigates the fracture mechanical properties of concrete, using crushed concrete aggregates (CCA) and granulated blast furnace slag (GGBS) for partial cement replacement. CCAs made from prefabricated concrete replace 100% of the fine and coarse fractions in concrete recipes with *w*/*c* ratios of 0.42 and 0.48. Two pre-treatment methods, mechanical pre-processing (MPCCA) and accelerated carbonation (CO_2_CCA), are investigated for quality improvements in CCA. The resulting aggregates show an increased density, contributing to an increase in the concrete’s compressive strength. The novelty of this paper is the superposition of the effects of the composite parts of concrete, the aggregate and the cement mortar, and their contributions to concrete fracture. Investigations are directed toward the influence of fine aggregates on mortar samples and the influence of the combination of coarse and fine aggregates on concrete samples. The physical and mechanical properties of the aggregates are correlated with mortar and concrete fracture properties. The results show that CCA concrete achieves 70% of the fracture energy values of concrete containing natural aggregates, and this value increases to 80% for GGBS mixes. At lower *w*/*c* ratios, MPCCA and CO_2_CCA concretes show similar fracture energies. CO_2_CCA fine aggregates are the most effective at strengthening the mortar phase, showing ductile concrete behavior at a *w*/*c* ratio of 0.48. MPCCA aggregates contribute to higher compressive strengths for *w*/*c* ratios of 0.42 and 0.48. Thus, mechanical pre-processing can be improved to produce CCA, which contributes to more ductile concrete behavior.

## 1. Introduction

The aggregates used in concrete are a finite resource. In Sweden, natural sand of fluvial origin is a critical resource which is necessary for the preservation of drinking water reservoirs. Therefore, Swedish law has placed restrictions on the volume of sand that can be extracted for concrete and road applications [1]. Alternative aggregates that fit concrete specifications must be identified to manage the increased demand for concrete production. The challenges of waste reduction and the extraction of natural aggregates may be resolved by using crushed concrete aggregates (CCAs) in concrete. About 2 million tons of concrete waste accumulates in Sweden annually which can be used to produce coarse and fine CCA fractions [2]. Resource-efficient concrete containing CCA replacement percentages as high as 100% is referred to as circular concrete.

Another important aim with respect to concrete is the reduction of the CO_2_ footprint associated with its production. This arises mainly from the manufacture of cement, which accounts for 5% of global anthropogenic CO_2_ emissions contributing to climate change [3,4]. The use of ground granulated blast furnace slag (GGBS) as a supplementary cementitious material (SCM) contributes to lowering the CO_2_ footprint of concrete. Concrete with a lowered CO_2_ footprint is climate-reduced concrete. The use of GGBS is shown to favor the development of strength in concrete at high CCA replacement percentages [5]. A circular and climate-reduced concrete is obtained by combining an alternative aggregate, CCA, with an alternative cement, GGBS. The focus of this paper is to investigate the resulting structural concrete for its mechanical and fracture mechanical properties.

**Figure 1:**
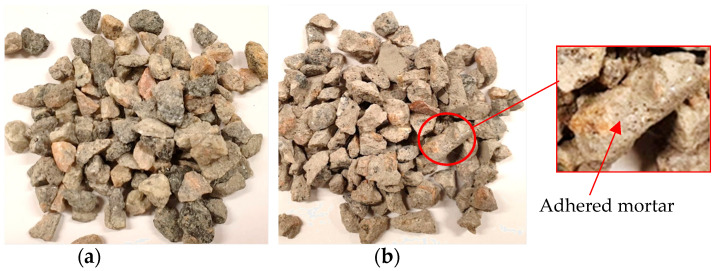
Coarse aggregate fractions; (**a**) crushed rock; (**b**) crushed concrete aggregates.

The CCA is a composite of aggregate and adhered cement mortar, unlike stone-based aggregates that are homogeneous [6]. Figure 1 shows a comparison of the coarse fractions of crushed rock and CCA. The CCA has an angular form similar to the crushed rock; however, it differs due to the mortar adhered to the CCA, also shown in the figure.

In most cases, CCAs show reduced densities compared to natural aggregates except for CCAs derived from high-strength concrete [6,7]. The use of a lower-density CCA results in mechanical properties inferior to those of concrete which contains natural aggregates. The removal of adhered mortar via mechanical pre-processing methods is proven to result in improvements in compressive strength which can be likened to natural-aggregate concrete [8]. The mechanical properties of concrete are also enhanced via the densification of the adhered mortar. Some investigated methods are the pre-treatment of CCA with a silica fume [9] and accelerated carbonation techniques [10]. In addition to the densification of adhered mortar, accelerated carbonation also contributes to the sequestration of CO_2_ from CO_2_ sources such as flue gas [11].

The reduced mechanical performance of CCA concrete is claimed to be due to the weaker interface formed between the cement paste and aggregate which is caused by adhered mortar [12]. Studies claim that pre-treatment methods such as mechanical pre-processing and accelerated carbonation improve the aggregate–mortar interface [9,11,13]. These studies used microstructure analyses to show interface changes before and after pre-treatments. However, further investigations and quantitative measurements are required.

**Figure 2:**
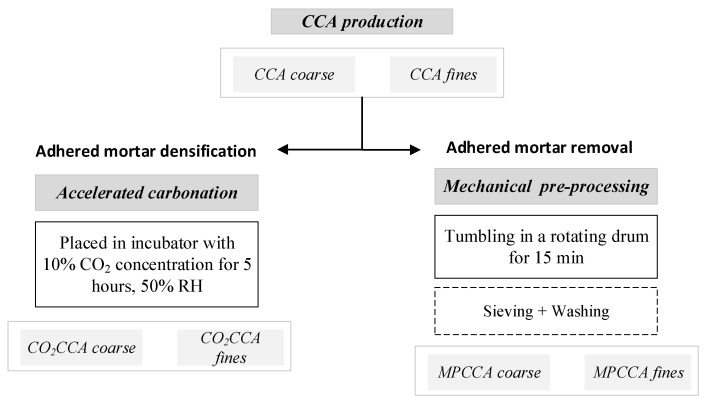
Pre-treatments for CCA densification.

Concrete rubble undergoes three crushing and sieving steps, resulting in fine and coarse CCA fractions. Mechanical pre-processing involves tumbling the CCA at 50 RPM for 15 min in a rotating ribbed drum to dislodge adhered mortar. The adhered mortar is removed by washing the aggregates on sieves [8]. The accelerated carbonation technique involves exposing CCA fractions to CO_2_ gas at a concentration of 10%, corresponding to the concentration of CO_2_ in flue gas from local incineration plants. A sealed incubator is prepared to maintain an exposure duration of 5 h at a relative humidity of 50%. The parameters for the carbonation setup are designed to maximize CO_2_ uptake and are based on previous research [10]. The schematics of performing mechanical pre-processing and accelerated carbonation techniques on coarse and fine CCA fractions are shown in Figure 2.

### Background

The toughness of concrete in tensile failure needs to be investigated to ascertain the concrete’s resistance to crack propagation. The fracture energy G_F_ is one such parameter for measuring tensile toughness [14]. The focus of this study is the determination of G_F_ through a Mode I failure based on the opening of a crack [15]. G_F_ measurements using three-point bending tests on notched concrete specimens are most commonly seen in the literature related to recycled aggregate concrete [16,17,18,19]. Alternatively, measurements of G_F_ can be made on notched cylindrical and cubic specimens of CCA concrete, as seen in [20]. The opening of the notch is achieved via the wedge splitting method, which was patented by the same author.

The fracture mechanical properties of concrete are influenced by the aggregate and cement mortar phases [21]. Coarse aggregates function as crack arrestors and therefore have a strong influence on the concrete’s fracture energy. The fracture energy is observed to increase in aggregates with increased particle densities. This varies with the type of aggregate [22,23] and the maximum aggregate size [24] and strength, which is measured via the aggregate crushing value [16]. The aggregate shape also influences the fracture energy such that concrete containing angular crushed aggregates shows a higher fracture energy than concrete containing spherical aggregates [25]. Since the claims about coarse aggregates are more stringent for high-strength concrete, investigations on the influence of coarse aggregates are mostly found in conjunction with high-strength concrete [22,24,26]. The fracture mechanical properties of CCA are mostly investigated for normal-strength concrete recipes, as seen in [16,17].

The cement mortar phase responds first under loading to produce microcracks. The strengthening of the cement mortar phase inhibits the initiation of cracks, thereby increasing the concrete’s fracture energy. This is achieved either via a reduction in the *w*/*c* ratio or with SCMs such as silica fume or GGBS [22,23,24,26]. Fine aggregates of crushed granite also contribute to strengthening the mortar phase at the same *w*/*c* ratio [27]. The angular shape and texture of crushed rock lead to aggregate interlock, which increases the concrete’s strength [27,28]. Aggregates with an increased content of fines (<0.075 mm) contribute to the densification of the mortar phase. This is seen in the case in which fine, crushed limestone aggregates replace river sand [29]. The grading of the fine fraction of the CCA after crushing has a closer resemblance to natural sand than crushed rock fines. Thus, there are technical gains when exchanging natural fines for recycled aggregates. Pre-treatments focused on adhered mortar removal, such as mechanical pre-processing, have been shown to improve the CCA grading, resulting in a stronger mortar phase in the concrete [30].

Fracture energy tests show a large variation in the results for replicates of the same concrete [17]. Tests on cement mortar samples provide a more robust assessment of an aggregate’s influence on concrete fracture. The fracture energy of mortar was tested via impact loading provided by a Charpy apparatus. The Charpy tests seen thus far in the literature are limited to mortar containing natural aggregates [31]. The impact modulus of toughness (the impact energy/unit volume of a specimen), as determined via the Charpy test, shows a good correlation with the compressive strength of cement mortar [31]. There have been very few investigations directed toward the fracture mechanics of cement mortar containing recycled aggregates. Akono et al. [32] determine the fracture toughness of mortar containing recycled aggregate fines using the microscopic scratch test. Their results showed a reduction in fracture toughness of 8% due to the increased porosity and decreased density of the mortar phase [32].

So far in the literature, only chemical-based pre-treatment methods have been investigated for their influence on the fracture properties of concrete containing CCAs [17,33,34]. This article aims to address this research gap by investigating the fracture energy of a concrete produced using a CCA that has been improved via mechanical pre-processing and accelerated carbonation. Analyses of the effects of both coarse and fine CCA replacements on concrete fracture properties are few in the literature [18,20,35], in which the influence of fine CCAs on fracture properties has not been addressed. This study focusses on a 100% replacement of the fine and coarse CCAs in concrete. The novelty of this paper and its contribution to the field are the superimposition of the effects of the composite parts of concrete, the aggregate and the cement mortar, and their contributions to concrete fracture. The investigations are directed towards resolving the fine aggregate’s influence on mortar samples and the influence of the combination of coarse and fine aggregates on concrete samples.

This study focuses on the 100% replacement of coarse and fine aggregates with a CCA. The aggregate’s influence is investigated using two CCA pre-treatment methods which produce fine and coarse CCAs of different qualities. Both pre-treatment methods result in concrete with a compressive strength comparable to natural-aggregate concrete. The influence of cement mortar is investigated by varying the *w*/*c* ratio of the concrete mixes. Two *w*/*c* ratios are: investigated 0.48 for normal-strength concrete and 0.42 for a high-strength concrete. A 30% replacement of cement with GGBS for a *w*/*c* equivalent 0.42 is also investigated. A reference concrete mix containing natural coarse and fine aggregates is made for all three recipes. The experimental scheme consists of tests on the physical, mechanical and fracture mechanical properties of the aggregates, cement mortar. and concrete. The number of test samples are aimed at understanding the dependence of concrete’s mechanical properties on aggregate and mortar strength. For a regular statistical assessment, more test samples are required. This study does not include any microscopic analyses.

## 2. Materials and Methods

The concrete waste was sourced from rejected prefabricated elements from an industrial facility. The crushed concrete aggregates (CCAs) were prepared by crushing concrete in a jaw crusher, followed by sieving the particles to obtain coarse and fine aggregate fractions. The aggregates were pre-treated via mechanical pre-processing and accelerated carbonation techniques according to the procedures in Figure 1; the aggregates are denoted MPCCA and CO_2_CCA, respectively.

### 2.1. Testing Aggregate Properties

The physical and mechanical properties of the coarse and fine aggregates were investigated. The particle size distribution of the coarse and fine aggregates was tested according to SS-EN 933-1:2012 [36]. The flakiness index for the coarse aggregates was determined using the standard SS-EN 933-3:2012 [37]. The flakiness index for fine aggregates up to 1 mm was investigated according to SBUF 122270 [38]. The coarse and fine aggregate fractions were tested for their apparent density and unit weight (bulk density), using SS-EN 1097-6:2013 [39] and ASTM C29/29M–17a [40], respectively.

The aggregate crushing value (ACV) was determined according to the standard BS-812-110:1990 [41]. The ACV, expressed as a percentage of the sample mass, is the mass of crushed fines that pass through a 2.6 mm sieve when an un-compacted coarse aggregate sample undergoes compressive loading [28]. The aggregate elastic modulus was determined together with the ACV in the same test, as shown in previous research [16]. The ACV specimen was loaded at a rate of 0.5 kN/s up to a maximum load of 400 kN. The strain at different loads was calculated from the changing deflection values relative to the original height of the aggregate’s sample. The elastic modulus was calculated as a secant on the stress–strain curve between the strain values 0.1 and 0.25 mm/mm for this study. Secants may be determined between 0 and 0.15 mm/mm, as seen in previous research [16]. The difference in the strain interval is to allow for specimen stabilization under loading, which occurs until 0.1 mm/mm.

### 2.2. Testing Cement Mortar

**Figure 3:**
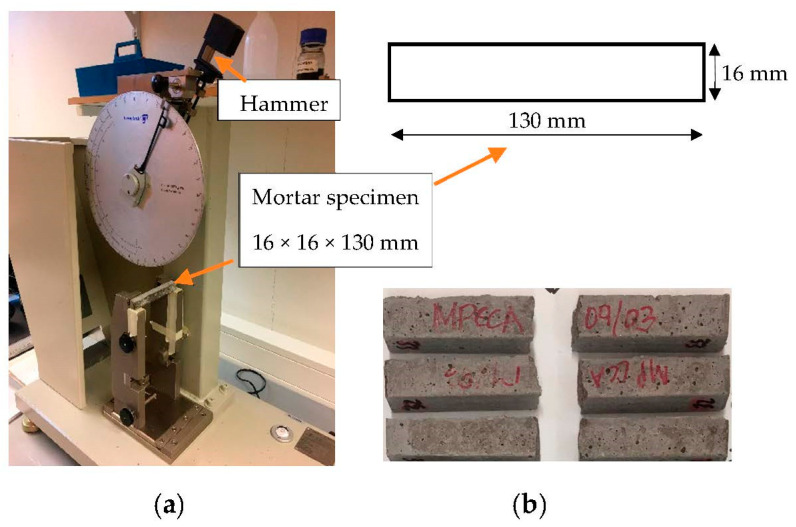
(**a**) Charpy testing apparatus and mortar specimen; (**b**) mortar specimens after fracture.

**Figure 4:**
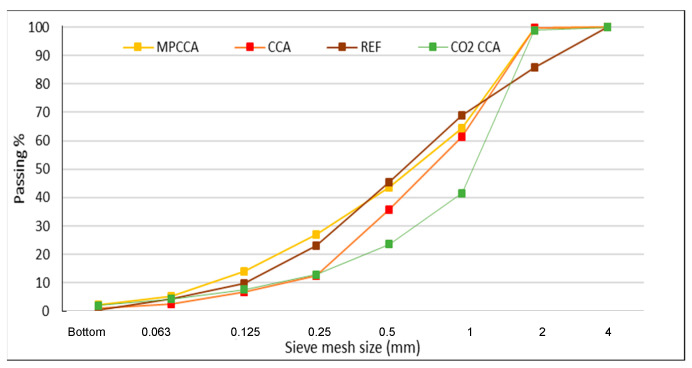
Fine aggregate grading curves for cement mortar samples.

The Charpy test shows the energy required to break a prism specimen using dynamic loading created by a swinging hammer connected to a pendulum, as shown in Figure 3. The weight of the hammer and the length of the pendulum depend on the size of the mortar specimen and the size of the aggregate. The hammer weighs 0.289 kg, and the length of the pendulum is 206 mm. The Charpy results can be interpreted as the load to failure of a mortar prism in N/m. The Charpy test was conducted according to the standard ISO 179-1:2010 [42], applied for cement mortar. The mortar prisms were of the size 16 × 16 × 130 mm. The maximum aggregate size was limited to 4 mm following the rule D/4 in which D is the width of the prism, in this case, 16mm. The reference mortar mix comprised a fine fraction of 0/4 mm. The CCA, MPCCA and CO_2_CCA mixes had two fine fractions, 0/4 and 0.5/4 mm, to match the grading of the reference mix. The grading curves of the fine aggregates are shown in Figure 4, and Table 1 shows the recipes for the mortar mixes.

The Charpy test has a dynamic loading character in comparison to the static three-point bending tests used to determine fracture energy on concrete specimens. Therefore, the Charpy test provides an over-estimated value for the mortar compared to the fracture energy of concrete. Compressive strength was tested on mortar samples measuring 40 × 40 × 160 mm, and the elastic modulus was tested using 100 × 200 mm cylinders, using the standards SS-EN 1015-11 [43] and SS-EN 12390-13 [44], respectively. These mechanical properties are evaluated for mortar specimens following normal concrete with a *w*/*c* ratio of 0.48.

### 2.3. Testing Concrete

The cement used was CEM II/A-LL 42.5 R manufactured by CEMENTA, Sweden, and the superplasticizer was polycarboxylate-based with a dry content of 24% by weight; the proportions are shown in Table 1. The GGBS was a commercial product, Merit 5000, which was produced in Sweden. The mix containing GGBS with a *w*/*c* equivalent to 0.42 is denoted 0.42 SLAG. The GGBS influences the workability of the concrete, as seen via the increased slump values which may influence the hardened concrete’s properties [45]. No specific adjustments were made for the rheological properties of the GGBS mixes. The reference concrete mix (REF) was composed of natural aggregates (NAs). The coarse and fine aggregates were crushed stone and river sand, respectively. The recycled aggregates showed gap-grading so as to match the grading curve of the natural aggregates (Figure 4). Therefore, the 0/8 mm natural aggregate fraction in the reference concrete was replaced by two fine CCA fractions of 0/4 and 0.5/4 mm. The natural and recycled aggregate fractions which were investigated along with their concrete mixes are shown in Table 1. The mixing water, absorption water and superplasticizer contents were the same for the mortar and concrete recipes, respectively.

**Figure 5:**
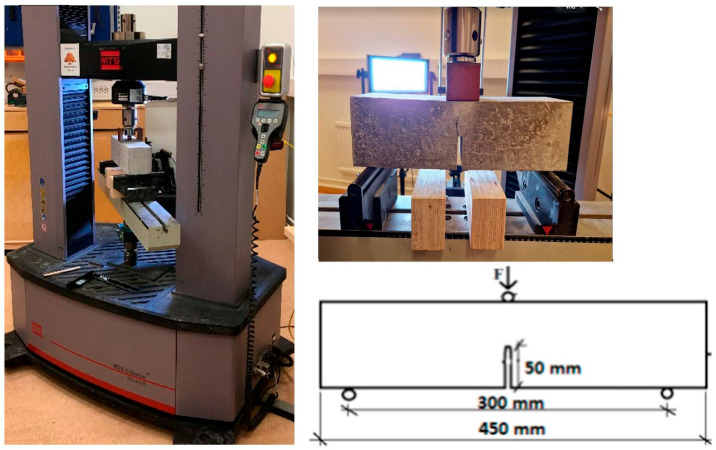
Testing fracture energy on notched concrete beams.

The compressive strength, elastic modulus and splitting tensile strength at 28 days were measured using three sets of 100 × 200 mm cylinders, according to SS-EN 12390-3 [46], SS-EN 12390-13 [44] and SS-EN 12390-6 [47], respectively. The elastic modulus was calculated as a secant on the ascending stress–strain curve between the stress value of 0.5 MPa and a second value corresponding to 30% of the compressive strength value. The fracture energy was tested using three-point bending tests on beams 350 mm long, with widths and depths of 100 × 100 mm, respectively, as shown in Figure 5. A notch with a thickness of 5 mm and a depth of 50 mm was sawn in the center of the beam just prior to testing to ensure a localized fracture. The experiments were performed using a uniaxial testing machine (MTS) with a load cell capacity of 100 kN. The deformation (δ) was measured in the center of the beam, using the crosshead movement. The loading was deformation-steered, maintaining a deformation of 1.8 mm/min at the center while ensuring that the peak load was reached within 60s. The load and deformation data were recorded until the beam separated in two halves.

The fracture energy G_F_ was calculated according to the method proposed by the RILEM technical committee 50, given by the formula $G_{F}=\frac{\left(W_{0}+mg\delta_{0}\right)}{A_{lig}}$ in N/m [48]. W_0_ represents the area under the load–deformation curve (N/m), δ_0_ is the deformation at the final fracture (m), m is the sum of the weight of the beam between the supports and the loading arrangement (kg), g is acceleration due to gravity 9.81 m/s^2^ and A_lig_ (m^2^) is the cross-sectional area of the beam above the notch.

## 3. Results

To understand the aggregate’s influence on the mechanical properties of mortar and concrete, the following results are reported in this chapter:

- The mechanical performance of the aggregates, reported using the elastic modulus;
- The properties of the fine aggregates, with the mortar’s mechanical and Charpy energy properties;
- The properties of the coarse aggregates, with the mechanical properties and fracture energy of the concrete.

### 3.1. The Physical and Mechanical Properties of the Coarse Aggregates

Tests of physical properties—the apparent density, flakiness index and unit weight—were conducted for both the coarse and fine fractions. The coarse and fine aggregates’ properties are presented separately in Table 2 and Table 3, respectively.

**Figure 6:**
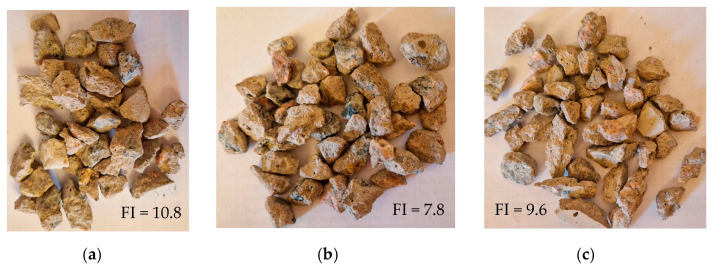
Photographs of aggregates: (**a**) CCA, (**b**) MPCCA and (**c**) CO_2_CCA; flakiness index values are provided in %.

The removal of adhered mortar manifested as a reduction in the flakiness index for the MPCCA aggregate. From the point of view of the aggregates’ shapes, this positions the MPCCA aggregate between CCA and natural coarse gravel. The MPCCA aggregate was densified via the removal of adhered mortar, while the CO_2_CCA aggregate was densified via the strengthening of the adhered mortar. The densities of the aggregates are reported in Table 2. Changes in the shape properties of the 8/11.2 fractions of the CCA, MPCCA and CO_2_CCA aggregates are shown along with flakiness index values in Figure 6.

**Figure 7:**
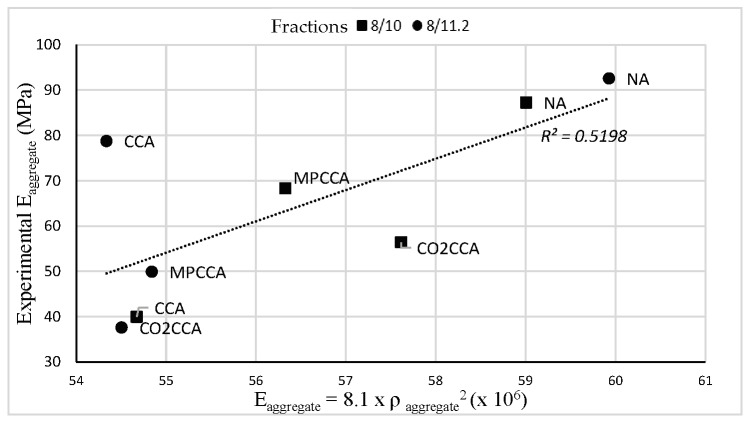
Elastic modulus of coarse aggregates.

The aggregate elastic modulus is related to the density of the aggregate. This is given by the Müller-Rochholz [49] relation shown in Equation (1).

(1) $$E_{aggregate}=8.1\times {\rho_{aggregate}}^{2}$$

where E_aggregate_ is the elastic modulus of the aggregate, and $\rho_{aggregate}$ is the apparent density of the aggregate. The calculated E_aggregate_ results are shown in Figure 7.

The improved densities of the MPCCA and CO_2_CCA aggregates should bring improvements to the s elastic modulus values. An aggregate’s elastic modulus has a strong influence on the stiffness of concrete and thus on the fracture mechanical properties of the concrete. In this way, improvements in the aggregate density can bring about improvements in the fracture mechanical properties of concrete.

In this study, the aggregate elastic modulus was determined experimentally via the confined axial loading of a constant volume of aggregates during an ACV test. The test results confirm that improvements in aggregate density result in an increase in the aggregates’ elastic modulus. The experimental and calculated elastic modulus are plotted against each other for coarse aggregate fractions of 8/10 and 8/11.2 in Figure 7.

The experimental elastic modulus shows a reasonable correlation with the elastic modulus calculated using aggregate density values for each of the investigated aggregate types. The best correlation is seen for NAs, followed by MPCCA aggregates. The natural aggregates with the highest density values also show the highest elastic modulus values during testing. The mechanically pre-processed aggregates show the most similar behavior to the natural aggregates (correlation factor: 0.9). The lowest in elastic modulus correlation is seen in the case of the CO_2_CCA aggregate, although there was an increase in density; values are provided in Table 2. This results in an increase in the concrete’s compressive strength, presented later in this article.

### 3.2. Physical Properties of Fine Aggregates

The properties of the fine aggregate fractions used in both the mortar and concrete mixes are shown in Table 3. Both the concrete and mortar mixes used the same recycled fine aggregate fractions of 0/4 and 0.5/4 mm. The natural aggregate fraction in the REF concrete is 0/8 mm, and in the REF mortar, the natural aggregate fraction is 0/4 mm.

**Table 3:** Properties of fine aggregates.

Properties	Fine Aggregate Fractions (mm)							
	0/4			0.5/4			0/4	0/8
	CCA	MPCCA	CO_2_CCA	CCA	MPCCA	CO_2_CCA	NA	NA
Apparent density (kg/m^3^)	2677	2718	2752	2656	2637	2639	2701	2701
Flakiness index (%)	5.90	5.2	5.6	6.5	5	5.08	1.8	4.5
Unit weight (kg/m^3^)	1415	1385	1567	1314	1268	1263	1748	1837

The density and unit weight of the fine recycled aggregate fraction of 0/4 mm improved markedly via accelerated carbonation compared to mechanical pre-processing. This effect is not seen for the 0.5/4 mm fraction as the fraction lacks fines which show the highest tendency for carbonation. For the MPCCA, the process of sieving and washing resulted in the loss of high-density fines. The increase in particle density of the CO_2_CCA fraction manifested as an increase in the unit weight, bringing it closer to the NA fraction.

### 3.3. Mechanical Properties and Charpy Energy of Mortar

The results of the mechanical properties of the mortar, such as its compressive strength (f_c_) and elastic modulus (E_mortar_), for mixes with a *w*/*c* ratio of 0.48 are shown in Table 4. The Charpy energy for the mortar mixes 0.48, 0.42 and 0.42 SLAG are seen in Table 5.

The fine aggregate density governs the compressive strength of the mortar. Improvements in the density of the CO_2_CAA fractions appear as an increase in compressive strength in the corresponding mortar mixes. The compressive strength of the mortar follows the same order as the fine aggregate density: CO_2_CCA > NA > MPCCA > CCA. The elastic modulus values of the mortar samples with recycled aggregates are much lower than that of the reference mix with NA.

**Figure 8:**
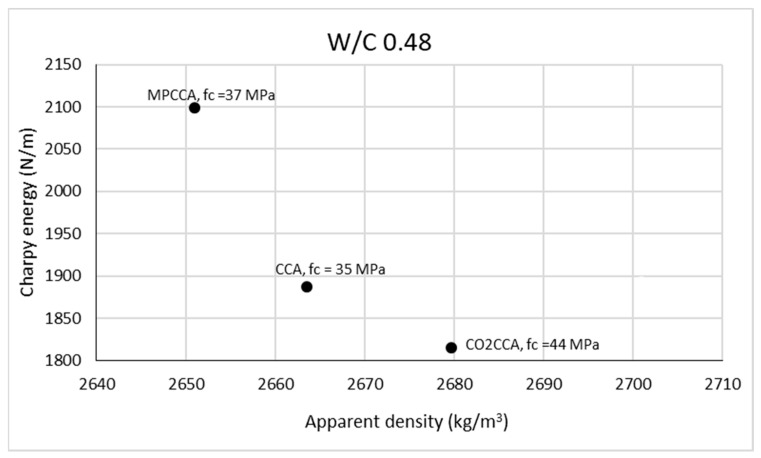
Relationship between the Charpy energy of mortar and the density of fine aggregates.

The potential energy of a hammer dropping from a height is translated into the energy required to break a specimen of a given cross-sectional measurement, as seen in the Charpy test. Previous studies showed an inverse linear relationship between the Charpy energy and the compressive strength of mortar [31]. Mortar mixes with higher compressive strengths result in lower values of Charpy energy due to brittle behavior. This can be explained via the aggregate density, which has a governing effect on compressive strength and thus on the Charpy energy. Figure 8 shows the results of the Charpy energy tests of mortar samples against the corresponding fine aggregate densities and compressive strengths of mortar specimens for a *w*/*c* ratio of 0.48.

For the *w*/*c* ratio of 0.48, there is a good correspondence between the Charpy energy and compressive strength test results which can be summarized as follows:

- Densified aggregates result in lower Charpy energy values;
- The Charpy energy has an inverse relationship to the compressive strength of mortar. Higher Charpy energies are observed for mortars with lower compressive strengths.

### 3.4. Mechanical and Fracture Mechanical Properties of Concrete

The compressive strength (f_c_), static elastic modulus (E_concrete_) and splitting tensile strength (f_t_) were tested for the concrete mixes 0.48, 0.42 and 0.42 SLAG; the values are presented in Table 6. The fracture energy (G_F_), peak load and maximum deflection results are shown in Table 7.

The fracture energy results in Table 7 show that the CCA concrete reached 70% of the reference concrete fracture energy values for the *w*/*c* ratios 0.42 and 0.48. This is consistent with previous research related to the fracture energy of concrete composed entirely of fine and coarse CCA fractions [18,20]. With the inclusion of GGBS, the CCA concrete reached 80% of the reference concrete’s G_F_ values. Due to its fineness, the GGBS contributed to the densification of the cement paste [5], but it also densified the adhered mortar in the CCA aggregates [50]. The MPCCA and CO_2_CCA concretes still showed lower fracture energy values than the CCA concrete. This may be because mechanical pre-processing removed the adhered mortar which was available for the GGBS to densify before pre-processing. The adhered mortar on the CO_2_CCA aggregates was already densified via carbonation, therefore leaving less surface for densification via the GGBS.

## 4. Analysis of Results

### 4.1. Effect of Aggregate Shape on Fracture Energy

**Figure 9:**
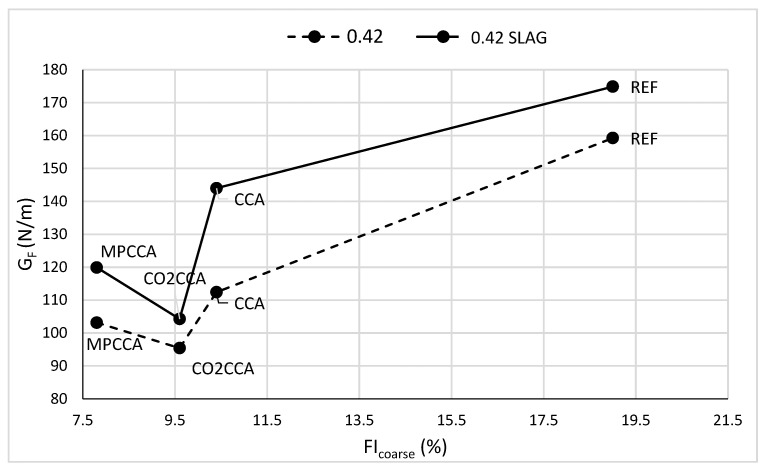
Relationship between the fracture energy of concrete and the flakiness index of a coarse aggregate.

Concretes comprised of angular coarse aggregates show higher fracture energies compared to concretes containing spherical aggregates for aggregates of similar densities [25]. Mechanical pre-processing removes the adhered mortar and also reduces the flakiness of recycled aggregates; flakiness index results are shown in Table 2. The relationship between the fracture energy, G_F_, and the flakiness index of the coarse aggregate, FI_coarse_, is shown for the 0.42 and 0.42 SLAG concrete mixes with a recycled coarse aggregate fraction of 8/11.2 mm in Figure 9. The 0.48 mix was not investigated as the flakiness index varies less among the 8/10 mm fractions from 7.7% for MPCCA to 9% for crushed stone in the REF mix.

There is a similar pattern between G_F_ and FI_coarse_ for both concrete mixes, indicating a relationship between fracture energy and an aggregate’s flakiness index. The REF mix containing crushed stone as a coarse aggregate shows the highest G_F_ and FI_coarse_ values amongst all the mixes, in addition to ductile behavior. The MPCCA aggregates show the lowest flakiness index and the concrete shows lowest G_F_ values and thus brittle behavior as it has lost the aggregate’s interlocking effect.

### 4.2. The Effect of the Elastic Modulus of an Aggregate on Concrete

The elastic modulus values of coarse aggregates and cement mortar contribute to the elastic modulus of concrete. Many models in previous research showed relationships between E_aggregate_, E_mortar_ and E_concrete_ for natural aggregates [28]. While investigating recycled aggregates from different sources, Butler et al. [16] showed that E_concrete_ increases with an increase in E_aggregate_ for coarse fractions. Investigations relating E_mortar_ with E_concrete_ for recycled aggregates are missing in the literature thus far.

**Figure 10:**
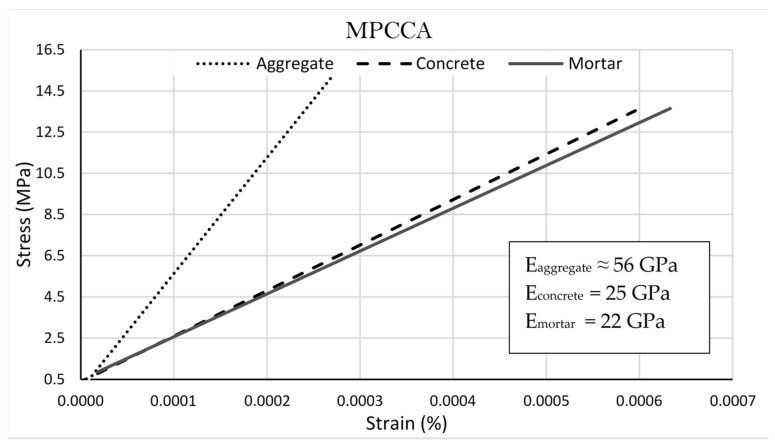
Stress–strain curves for aggregate, mortar and concrete for *w*/*c* 0.48 with MPCCA.

A graphical understanding of the influences of E_aggregate_ and E_mortar_ on the elastic modulus of concrete, E_concrete_, is shown via an arrangement of their respective stress–strain curves. The stress–strain curve for concrete is located in between the curves of the aggregate and mortar, respectively, as seen in the literature for natural-aggregate-based mortar and concrete in Neville, A.M. [21]. A similar representation is shown for MPCCA recycled aggregates in Figure 10.

The experimental and calculated elastic modulus values correspond well, particularly for the MPCCA-based mixes. The E_aggregate_ value assumed for the MPCCA aggregate is about 56 GPa from Figure 7. The aggregate’s stress–strain curve was constructed based on the calculated/experimental elastic modulus values of the aggregates. The elastic modulus value of a concrete containing recycled aggregates is thus represented as a function of both the elastic moduli of the aggregate and mortar phases, respectively. Similar inferences can be made for concrete and mortar mixes with aggregates treated via the accelerated carbonation technique (CO_2_CCA).

### 4.3. Fracture Energy

**Figure 11:**
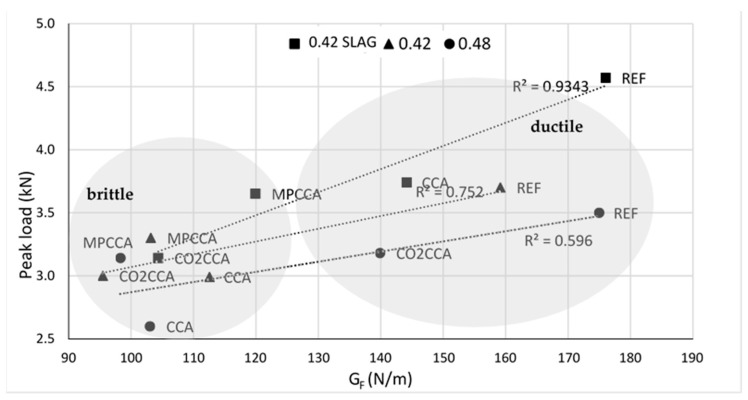
Correlation between the peak load and fracture energy of concrete; average values are shown.

The fracture energy, G_F_, was calculated as the area under the load–deflection curve resulting from a three-point bending test on concrete beams. High G_F_ values can result either from high peak load values or gradual and wide descending branches of the load–deflection curve. The G_F_ values were analyzed against the corresponding peak loads and time to failure. The fracture energy shows a better correlation with the corresponding peak loads compared to the time to failure; the peak loads are plotted against G_F_ in Figure 11.

The best correlation is seen for the concrete mix containing GGBS which behaved almost like a high-strength concrete, and the second-best correlation is shown by the 0.42 mix. The GGBS mix resulted in the highest peak load with the lowest time to failure, indicating more brittle behavior compared to the other mixes. This is also confirmed by the Charpy energy values in Table 5, which are the highest for the mortar mixes containing GGBS, especially for the MPCCA, CO2CCA and CCA aggregates.

#### The Aggregate’s Effect on Fracture Energy

**Figure 12:**
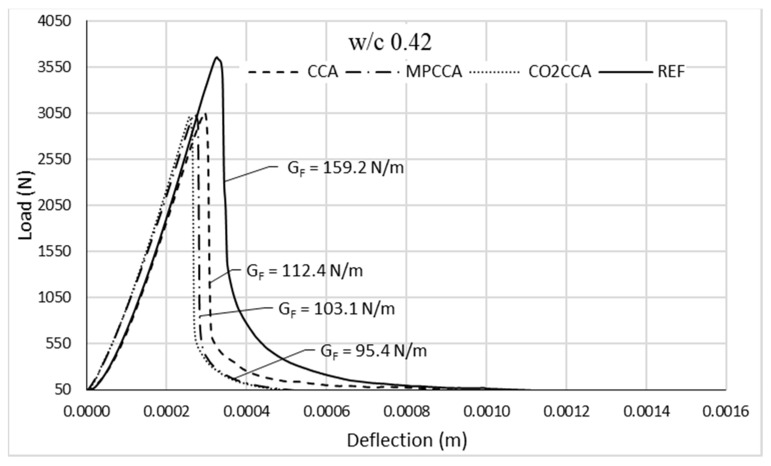
Load-deflection curves for concrete mixes with a *w*/*c* ratio of 0.42.

The aggregate’s effect on G_F_ is shown in Figure 12, using the average load–deflection curves for the 0.42 concrete mixes containing recycled aggregates. It can be seen that all the recycled aggregates yielded concrete samples with G_F_ values that were lower than the reference concrete, independent of the pre-treatment method used. This is a conclusive finding for concrete comprised of 100% recycled aggregates.

### 4.4. The Aggregate’s Influence on Compressive Strength

**Figure 13:**
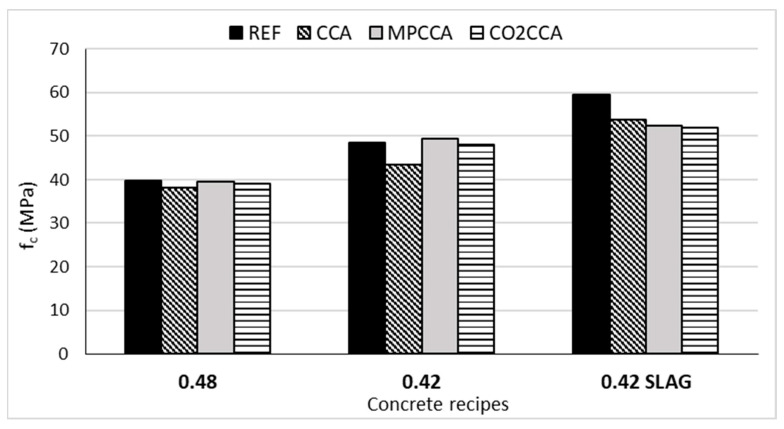
Compressive strength results for concrete mixes.

Concrete’s compressive strength is an outcome of the strengths of both the aggregate and cement paste phases [28]. From the perspective of the aggregate, coarse aggregates provide aggregate strength, while fine aggregates contribute to strengthening the cement mortar. Both mechanical pre-processing and accelerated carbonation were effective at achieving reference concrete strengths at ratios of 0.48 and 0.42 *w*/*c*, Figure 13. The addition of GGBS contributed to the strengthening of the cement mortar phase. The results of the mortar enrichment are more effective than the improvements to the aggregates resulting from the different pre-treatments. Therefore, CCA concrete has a higher compressive strength than concrete containing MPCCA and CO_2_CCA aggregates. A higher compressive strength does not imply a higher fracture energy, as pointed out in [16,24]. The MPCCA and CO_2_CCA concrete samples show lower G_F_ values than the reference concrete, including brittle failure at lower *w*/*c* ratios, while achieving the reference concrete’s compressive strength. This is unlike the CCA concrete, which does not meet the compressive strength of the reference concrete yet shows a higher fracture energy value than the MPCCA and CO_2_CCA concrete samples. Aggregates which received accelerated carbonation pre-treatment (CO_2_CCA) produce mixes that demonstrate ductile behavior at higher *w*/*c* ratios.

## 5. Conclusions

This study is an assessment of the contributions of aggregates and cement mortar to the fracture mechanical properties of concrete. CCA replaced 100% of the aggregates in the investigated concrete recipes. Two quality improvement methods were investigated for the recycled aggregates: mechanical pre-processing and accelerated carbonation. The resulting aggregates are denoted MPCCA and CO_2_CCA, respectively. Two *w*/*c* ratios were investigated, 0.42 and 0.48, including a climate-reduced concrete with GGBS with a *w*/*c* equivalent 0.42. Recommendations for normal and high-strength recycled aggregate concrete are made using the results of fracture mechanical investigations. This was achieved through a combined analysis of the aggregates’ properties, Charpy test results on mortar and mechanical properties and the fracture mechanical properties of the concrete. The conclusions of this study are as follows:

- The CCA concrete achieves 70% of the fracture energy value of the reference concrete independent of the *w*/*c* ratio, and this value increases to 80% for mixes with the addition of GGBS.
- Mortar mixes containing aggregates treated with accelerated carbonation, CO_2_CCA showed high compressive strength values, low Charpy energy values and ductile behavior. Concrete mixes containing CO_2_CCA aggregates showed lower fracture energy values than the reference concrete. Thus, CO_2_CCA aggregates fit concrete with high *w*/*c* ratios.
- The MPCCA concrete showed a lower fracture energy than the reference concrete due to the loss of the aggregate’s flakiness following mechanical pre-processing. Mixes containing mechanically pre-processed aggregates show more brittle behavior and higher compressive strength values which fit normal–high-strength concrete.

## Figures and Tables

**Table 1:** Recipe proportions for concrete and mortar mixes.

Mixes	0.48 CEM II				0.42 CEM II				0.42 SLAG 30% GGBS, CEM II			
Concrete and Mortar Mix Series	REF	CCA	MP CCA	CO_2_CCA	REF	CCA	MP CCA	CO_2_CCA	REF	CCA	MP CCA	CO_2_CCA
**Concrete Recipes (kg/m^3^)**												
Aggregate type	NA	CCA	MP CCA	CO_2_ CCA	NA	CCA	MP CCA	CO_2_ CCA	NA	CCA	MP CCA	CO_2_ CCA
8/10	729	708	708	708	-	-	-	-	-	-	-	-
8/11.2	-	-	-	-	729	728	728	728	729	728	728	728
0/8	845	-	-	-	845	-	-	-	845	-	-	-
0.5/4	-	550	157	550	-	530	141	530	-	530	141	530
0/4	-	314	708	314	-	314	703	314	-	314	703	314
Water absorption water	-	44	36	37	-	44	36	37	-	44	36	37
CEM II	490				490				343			
GGBS (k = 0.8)	-				-				149			
Mixing water	234				205.8				194.1			
Superplasticizer	3.7				5				3.7			
**Mortar Recipes (kg/m^3^)**												
Aggregate type	NA	CCA	MP CCA	CO_2_ CCA	NA	CCA	MP CCA	CO_2_ CCA	NA	CCA	MP CCA	CO_2_ CCA
0.5/4	-	1008	283	1008	-	1001	1310	1001	-	1001	1310	1001
0/4	1574	566	1291	566	1574	572	264	572	1574	572	264	572

**Table 2:** Properties of coarse aggregates.

Properties	Fraction 8/10 *w*/*c* 0.48				Fraction 8/11.2 *w*/*c* 0.42			
	NA	CCA	MP CCA	CO_2_CCA	NA	CCA	MP CCA	CO_2_CCA
Apparent density, ρ_aggregate_ (kg/m^3^)	2699	2598	2637	2667	2720	2590	2602	2594
Flakiness index (%)	9.51	8	7.7	8	19	10.4	7.8	9.6
ACV (%)	27	29	30	30	26	24	29	27
Unit weight (kg/m^3^)	1529	1267	1298	1295	1487	1326	1349	1319

**Table 4:** Mechanical properties of mortar mix *w*/*c* 0.48.

Mortar Properties	Mortar Mixes			
	REF	CCA	MPCCA	CO_2_CCA
f_c_ (MPa)	43.9	35.1	37.8	44.8
E_mortar_ (GPa)	29.2	23.7	22.0	22.0

**Table 5:** Charpy test results for cement mortar specimens.

Charpy Energy (N/m)											
0.48				0.42				0.42 SLAG			
REF	CCA	MP CCA	CO_2_ CCA	REF	CCA	MP CCA	CO_2_ CCA	REF	CCA	MP CCA	CO_2_ CCA
1893	1888	2099	1816	1877	1855	1959	1865	1974	1885	1984	1899

**Table 6:** Mechanical properties of concrete.

	f_c_ (MPa)				E_concrete_ (GPa)				f_t_ (MPa)			
	REF	CCA	MP CCA	CO_2_CCA	REF	CCA	MP CCA	CO_2_CCA	REF	CCA	MP CCA	CO_2_CCA
0.48	39.7	38.2	39.6	39.1	26.9	22.6	25.2	25.5	3.2	4.1	3.0	3.2
0.42	48.4	43.3	49.4	48.0	29.7	23.4	23.3	23.8	3.1	4.0	3.3	2.9
0.42 SLAG	59.6	53.7	52.4	52.0	36.4	27.1	29.5	29.4	4.4	3.1	4.4	3.9

**Table 7:** Fracture mechanical properties of concrete.

Fracture Parameters	Concrete Mixes											
	0.48				0.42				0.42 SLAG			
	REF	CCA	MP CCA	CO_2_CCA	REF	CCA	MP CCA	CO_2_CCA	REF	CCA	MP CCA	CO_2_CCA
Peak load P_max_ (kN)	3.50	2.60	3.14	3.18	3.70	2.99	3.30	3.00	4.57	3.74	3.65	3.14
Deflection δ_0_ × 10^−3^ (mm)	2	1.4	0.9	2	1.5	1.5	0.9	0.9	1.1	1.3	1.1	1.5
Fracture energy GF (N/m) $G_{F}=\frac{W_{0}+{mg\delta_{0}}_{}}{A_{lig}}$	174.87	103.01	101.77	140.01	159.26	112.44	103.16	95.43	175.95	144.04	119.91	104.35
Time to failure (s)	463	156.3	320.7	459.7	344.3	310.0	150.7	162.6	212.7	267.7	203.7	333.6

## Data Availability

All research data is provided in the results.
